# Metabolic dysfunction and inflammatory disease: the role of stromal fibroblasts

**DOI:** 10.1111/febs.15644

**Published:** 2020-12-17

**Authors:** Hussein Farah, Stephen P. Young, Claudio Mauro, Simon W. Jones

**Affiliations:** ^1^ Institute of Inflammation and Ageing MRC‐Versus Arthritis Centre for Musculoskeletal Ageing Research University of Birmingham UK

**Keywords:** fibroblasts, inflammation, innate immune cells, metabolism

## Abstract

Mesenchymal stromal fibroblasts have emerged as key mediators of the inflammatory response and drivers of localised inflammation, in part through their interactions with resident and circulating immune cells at inflammatory sites. As such, they have been implicated in a number of chronic inflammatory conditions as well as in tumour progression through modifying the microenvironment. The connection between metabolic changes and altered phenotype of fibroblasts in inflammatory microenvironments has clear implications for our understanding of how chronic inflammation is regulated and for the development of new anti‐inflammatory therapeutics. In this review, we consider the evidence that changes to fibroblast metabolic state underpin chronic inflammation. We examine recent research on fibroblast metabolism in inflammatory microenvironments and consider their involvement in inflammation, providing insight into the role of fibroblasts and metabolism in mediating inflammatory disease progression namely cancer, arthritis and fibrotic disorders including chronic kidney disease, pulmonary fibrosis, heart disease and liver disease.

## Graphical abstract

Stromal fibroblasts are central mediators of chronic inflammatory disease. Changes to fibroblast metabolism (including glycolysis, oxidative phosphorylation, pentose phosphate pathway, glutamine metabolism and lactate transport) fuels and underpins the switch in fibroblast phenotype towards an inflammatory‐activated phenotype that drives chronic inflammation. Cancer‐associated fibroblasts drive tumour progression, activated synoviocytes drive joint inflammation, and activated myoblasts drive fibrotic disorders including chronic kidney disease, pulmonary fibrosis, heart disease and liver disease.

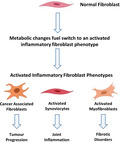

**Abbreviations**
CAF: cancer‐associated fibroblast
CKD: chronic kidney disease
ECM: extracellular matrix
FAO: fatty acid oxidation
GLS1: glutaminase‐1
HCC: hepatocellular carcinoma
HK2: hexokinase 2
HSC: hepatic stellate cells
MCT4: monocarboxylate transporter 4
MMP: matrixmetalloprotease
OA: osteoarthritis
PDK: pyruvate dehydrogenase kinase
PFKB: phosphofructo‐2‐kinase/fructose‐2,6‐bisphosphatase 3 enzyme
PGK1: phosphoglycerate kinase 1
PHGDH: phosphoglycerate dehydrogenase
RA: rheumatoid arthritis
SASP: senescence‐associated secretory phenotype
SHMT2: serine hydroxymethyltransferase 2
TCA: tricarboxylic acid cycle
TFAM: mitochondrial transcription factor A
TGF‐β1: transforming growth factor beta 1
TNF‐α: tumour necrosis factor α
Tregs: regulatory T cells

## Introduction

Fibroblasts are spindle‐shaped mesenchymal stromal cells that are found in a wide variety of parenchymal tissues. Primarily, they are believed to contribute to the homeostasis of the extracellular matrix (ECM), being capable of synthesising collagen, glycosaminoglycans, proteoglycans and other ECM proteins, thereby helping to maintain the structural architecture of tissues [1].

**Fig. 1:**
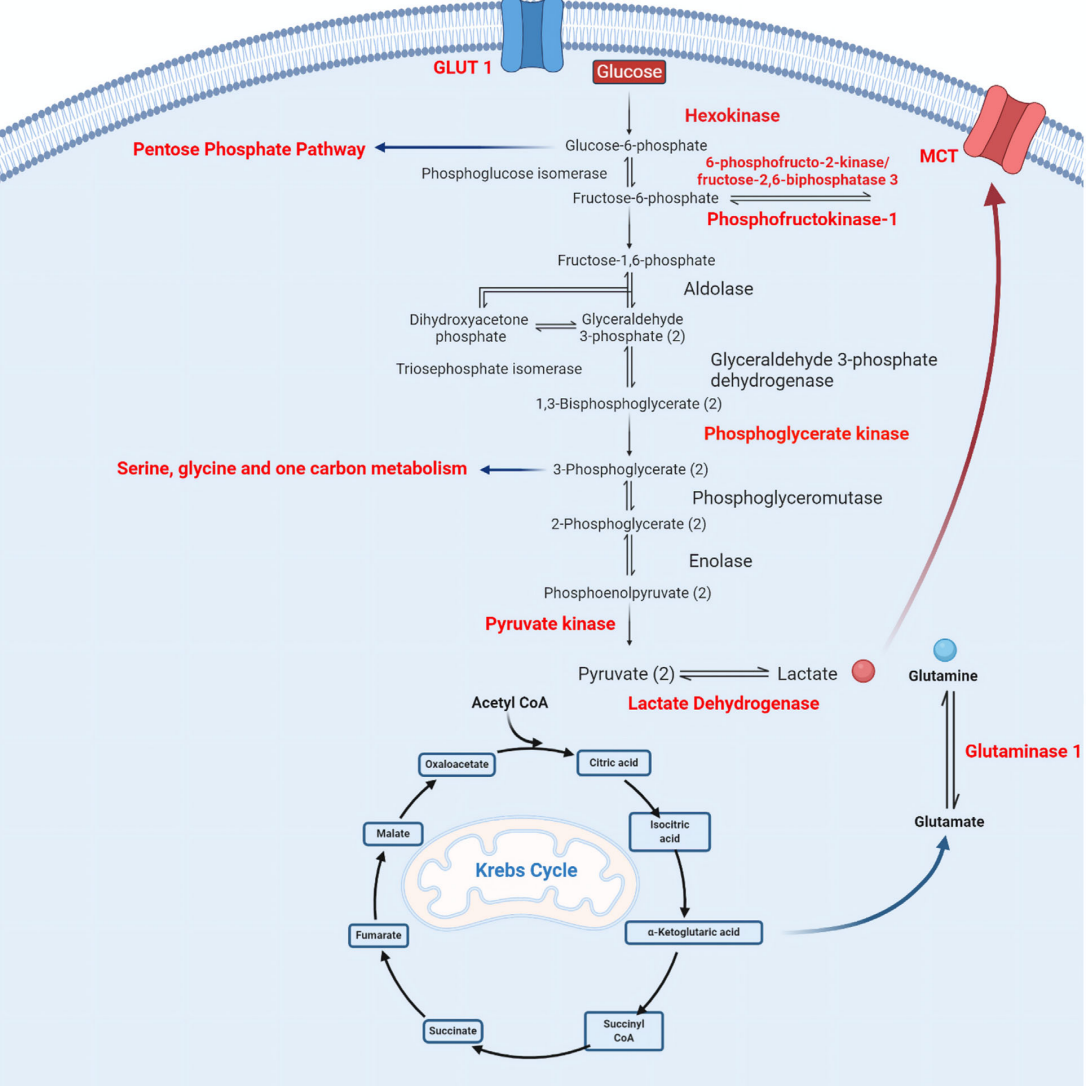
Dysregulated metabolic pathways of activated stromal fibroblasts. Fibroblasts in their nonactivated quiescent state maintain relatively high levels of energy production using glycolysis. However, activation via environmental stimuli such as inflammation results in an altered metabolic phenotype common to multiple fibroblast pathologies that increases key metabolic pathways including glycolysis, oxidative phosphorylation, lactate production, pentose phosphate pathway and glutamine metabolism.

However, in inflammatory microenvironments, there is now evidence that fibroblasts undergo major changes in cellular phenotype, switching to a more active, invasive and inflammatory phenotype. As a result, fibroblasts have been implicated as central mediators of tumour progression and a number of chronic inflammatory disorders including fibrosis and inflammatory joint pathologies [2]. A key driver of the phenotypic change of fibroblasts within inflammatory environments is now believed to be changes in the metabolic state of the cell. In normal physiological conditions, quiescent mesenchymal stromal fibroblasts exhibit a relatively high metabolic rate, compared to other cell types, maintaining substrate utilisation and ATP generation via glycolysis, pentose phosphate pathway and the tricarboxylic acid cycle (TCA) [3]. This high metabolic activity has been attributed to the state of continuous degradation and synthesis of macromolecules and membrane components through autophagy, the detoxification of free radicals through generation of increased NADPH for glutathione and thioredoxin and the maintenance of ECM via biosynthesis of ECM proteins [3, 4]. However, in inflammatory disease pathologies, there is now evidence that fibroblasts adopt an activated, highly metabolic phenotype, which is associated with disease progression by contributing to the inflammatory microenvironment through secretion of pro‐inflammatory cytokines and structurally remodelling ECM to facilitate aberrant cellular invasion and growth (Fig. 1). Indeed, in inflammatory joint conditions like rheumatoid arthritis (RA), activated synovial joint fibroblasts are recognised as central contributors of the invasive, degradative and highly inflammatory pannus–cartilage junction microenvironment. It has been proposed that this inflammatory‐activated fibroblast phenotype is fuelled by an upregulation of the aerobic glycolysis pathway, providing rapid generation of both ATP and biosynthetic building blocks required by proliferative biosynthetic cells.

## Cancer‐associated fibroblasts and tumour progression

There is now a large body of evidence for the role of both the tumour microenvironment and metabolism in supporting tumour progression and in drug resistance. Indeed, the use of alternative carbon sources as well as metabolic support and metabolite exchange between stroma and tumour cells termed the ‘reverse Warburg’ effect is now being discussed as a means of energy sustenance for cancer cells in the tumour environment [5, 6]. Therefore, understanding the role of metabolism in mediating the phenotype of cancer‐associated fibroblasts (CAFs), which are amongst the most abundant cell type in the tumour microenvironment, may have important implications with regards to identifying new therapeutic stratagems.

Derived from mesenchymal stromal cells, CAFs adopt an activated fibroblast phenotype not dissimilar to the phenotype of myofibroblasts present in inflammatory and fibrotic conditions. In this active phenotype, they promote tumour growth and invasion by supporting remodelling of the ECM via the production of ECM components such as type I and type II collagen as well as fibronectin [7, 8, 9, 10]. Importantly, CAFs also promote angiogenesis and invasion by contributing to the tumour inflammatory microenvironment. For example, CAFs secrete pro‐inflammatory cytokines and chemokines that recruit innate immune cells, including macrophages [11], monocytes [12] and neutrophils [13], to the tumour environment. Furthermore, CAFs also participate in antigen cross‐presentation [14], which promotes CD4+ T‐cell activation and reduction of CD8+ T cells [15, 16] and can promote invasion through activation of IL‐1 signalling [17].

Importantly, there is now increasing evidence that the activated, tumour‐promoting, phenotype of CAFs is underpinned by changes in metabolic activity. During their differentiation, CAFs upregulate the glycolytic enzymes hexokinase 2 (HK2) and phosphofructokinase (PFKL) in order to support the shift to glycolysis for rapid ATP generation [18]. The increase in HK2 in turn increases p27 protein expression via α‐ketoglutarate. Since cyclin‐dependent kinase 2 is inhibited by p27, this results in activation of the G1/S checkpoint, suggesting a link between fibroblast metabolic status and cell cycle [18]. Furthermore, the increased glycolytic activity of CAFs leads to greater lactate production [19], which together with an increase in the lactate exporting monocarboxylate transporter 4 (MCT4) [19] provides a greater source of lactate to cancer cells. Critically, despite being dismissed for many years as a waste product, lactate is now considered to be a key energy source for tumour cells to fuel oxidative phosphorylation [19, 20]. Indeed, blocking MCT transporters provides a possible avenue for disrupting CAF cancer metabolic symbiosis with genetic ablation of MCT4 expression overcoming adaptive resistance of cancer drug therapies [21].

Another aspect of CAFs metabolic reprogramming is the downregulation of the TCA cycle, with decreased isocitrate dehydrogenase 3α (IDH3α) observed in transforming growth factor beta 1 (TGF‐β1)‐induced differentiation of CAFs [22]. In this study, knockdown of IDH3α in primary fibroblasts resulted in increased glucose uptake and lactate production with concomitant decrease in oxygen consumption, whereas overexpression reduced TGF‐β1‐induced basal levels of glucose uptake and lactate production as well as increasing basal oxygen consumption [22]. In addition, CAFs are also reported to exhibit increased levels of glutamine and ketone bodies, both of which could provide metabolites required for oxidative phosphorylation in cancer cells [21]. Indeed, it has been postulated that glutamate is secreted into tumour microenvironment by cancer cells, is internalised by CAFs and recycled into glutamine for uptake by cancer cells for glutaminolysis and nucleotide production [23]. To this end, it has been shown that glutamine anabolic pathways are increased in CAFs from ovarian adenocarcinoma patients, compared to normal ovarian fibroblast [23].

It is currently unclear what the trigger is for the metabolic reprogramming exhibited by CAFs. However, interestingly, a reduction in the mitochondrial transcription factor A (TFAM) in stromal fibroblasts was reported to cause mitochondrial dysfunction and metabolic reprogramming consistent with the CAF‐activated metabolic phenotype of increased glycolysis and overproduction of lactate [24]. Notably, these TFAM‐deficient fibroblasts promoted tumour growth in a human breast cancer xenograft model and increased the mitochondrial activity of adjacent epithelial cancer cells [24].

## Fibroblast metabolism in chronic inflammatory disorders

### Synovial fibroblasts and inflammatory joint disease

Synovial fibroblasts are amongst the most important and abundant stromal cells at the joint synovium and contribute to the pathology of RA and other inflammatory joint diseases including osteoarthritis (OA), juvenile idiopathic arthritis, ankylosing spondylitis and psoriatic arthritis. Located at the synovial sublining and the lining layer, they are responsible for establishing cartilage integrity and the lubrication of the synovial joint by secreting ECM components into the synovial fluid. However, in RA, synovial fibroblasts adopt an inflammatory and invasive phenotype. RA synovial fibroblasts are hyperplastic and proliferate faster than non‐RA synovial fibroblasts, resulting in the formation of pannus‐like structures as they lose contact inhibition, become anchorage‐independent and upregulate expression of collagenases such as matrix metalloproteases and aggrecanases including ADAMTS4/5 that promotes cartilage degradation and perpetuate inflammation of the joint [25]. A similar inflammatory phenotype has recently been reported in OA, particularly in individuals who are obese. Obese OA synovial fibroblasts were more proliferative and exhibited a more inflammatory transcriptomic phenotype, compared to either normal‐weight OA patient fibroblasts or to non‐OA diseased fibroblasts [26].

Synovial fibroblasts are also innate immunity effectors as they express a number of pattern recognition receptors such as Toll‐like receptors 1‐7, retinoic acid‐inducible gene I and melanoma differentiation‐associated protein 5 [27, 28]. Exogenous peptidoglycan as well as endogenous ligands such as RNA from necrotic cells, hyaluronan, heat‐shock protein and fibrinogen is present in the RA synovial joint and can potentially activate synovial fibroblasts through TLR2 and TRL4 signalling and upregulate the expression of pro‐inflammatory cytokines and chemokines [29]. Stimulation of TLR3 with Poly(I:C) ligand along with tumour necrosis factor α (TNF‐α) and IL‐1β in synovial fibroblasts resulted in a cytokine secretome similar to RA synovial fluid, suggesting that the synovial fibroblast contributes considerably to the inflammatory environment at the RA synovial joint [29]. Indeed, synovial fibroblasts are major contributors to the inflammatory joint environment in both OA and RA disease through the release of pro‐inflammatory cytokines. In OA, the increase in synovial fluid concentrations of TNF‐α, IL‐6 and IL‐8 in obese patients is attributed to the increased secretion of these cytokines from obese synovial fibroblasts [30].

**Fig. 2:**
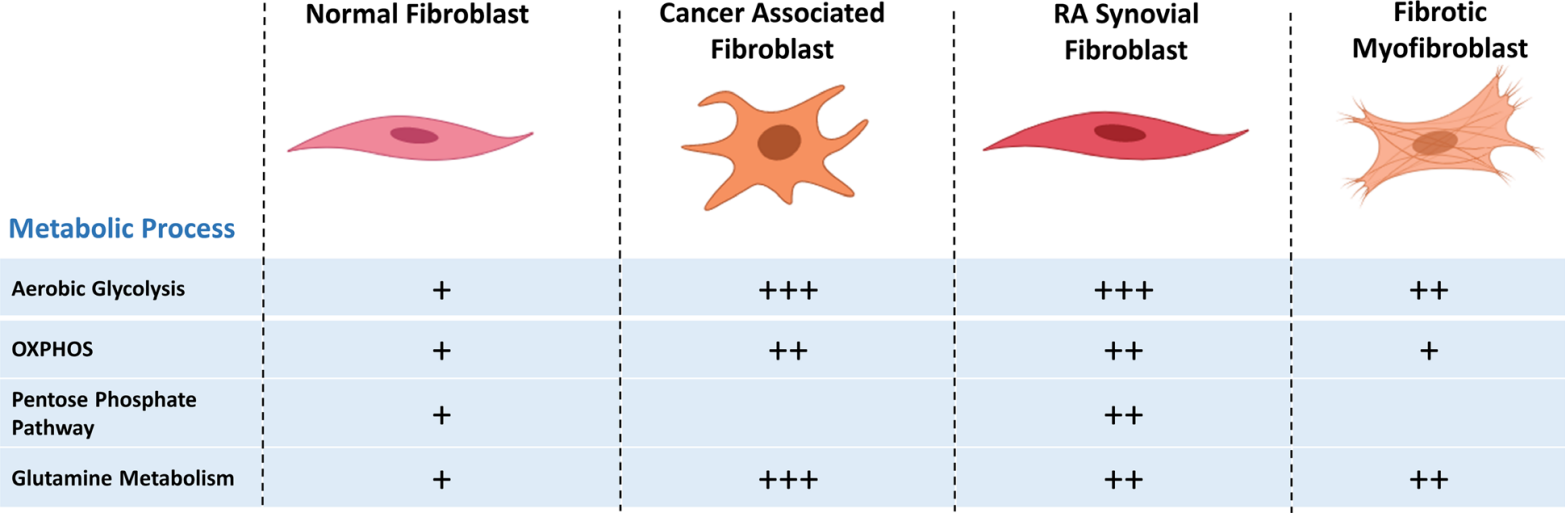
Commonality in metabolic signatures of activated stromal fibroblasts. Activation of stromal fibroblasts in multiple inflammatory pathological conditions, including CAFs, RA synovial fibroblasts and fibrotic myofibroblasts in lung, renal and cardiopathy disorders, results in an altered metabolic state. Changes occur in key metabolic processes including upregulation of the pentose phosphate pathway, aerobic glycolysis, glutamine metabolism and oxidative phosphorylation in CAFs and synovial fibroblasts. + symbols represent the strength of evidence and degree of pathway activation.

As in other inflammatory microenvironments, there is now growing evidence that synovial fibroblasts switch to a highly metabolically active state in the joints of patients with inflammatory disease. In Vasquez’ model, a key hallmark of rapidly proliferating mammalian cells is a shift towards aerobic glycolysis and a reduction in mitochondrial respiration when glucose uptake is not the rate‐limiting factor providing an energetically favourable catabolic state that solves the inherent physicochemical constraint of molecular crowding [31]. This switch from a resting regulatory state to a highly metabolically active state provides the macromolecules such as bioactive metabolites and nucleic acid intermediates required to promote and sustain the hyperplastic and hyperinflammatory state (Fig. 1). GC/TOF‐mass spectrometry‐based metabolomic profiling of RA synovial fibroblasts has revealed an alteration in sugar and amino acid metabolism as well as lipolysis as related to synovial hyperplasia and inflammation, providing further evidence of a link between aberrant metabolism and inflammation in RA synovial fibroblasts [32]. This change in basal glucose metabolism has also been observed in other studies and stimulation with pro‐inflammatory cytokines has been shown to upregulate glycolysis in RA synovial fibroblasts, similarly to that observed in CAFs (Fig. 2) [32, 33]. Glucose starvation reduced the proliferation and migration of RA synovial fibroblasts and decreased the levels of the pro‐inflammatory cytokine IL‐6 and degradative enzyme matrixmetalloprotease (MMP)1 and MMP3 [33]. Key glycolytic enzymes like phosphoglycerate kinase 1 (PGK1) and phosphofructo‐2‐kinase/fructose‐2,6‐bisphosphatase 3 enzyme (PFKFB) play a critical role in the RA synovial fibroblast phenotype. High levels of glycolytic enzyme PGK1 were observed in the blood and synovial tissue of RA patients and silencing of PGK1 in RA synovial fibroblasts using anti‐PGK1 siRNA reduced the secretion of pro‐inflammatory cytokines IL‐1β and IFN‐γ [34]. Inhibition of fructose 2,6‐bisphosphate production resulted in reduced levels of IL‐6 and the invasive phenotype of RA synovial fibroblasts [35, 36]. Inhibiting PFKFB3 glycolytic enzyme in RA synovial fibroblasts with 3PO (dipyridinyl‐propenone), a small molecule compound, which selectively inhibits PFK‐2 activity of the PFKFB3 enzyme, reduced fructose‐2,6‐bisphosphate and lactate levels as well as reducing the secretion of pro‐inflammatory cytokines [36]. Alongside this, silencing of TNFα with RNAi treatment decreased expression of glucose transporter glucose transporter 1 and glyceraldehyde 3‐phosphate dehydrogenase [36], providing further evidence of the connection between the metabolic state of the cell and the inflammatory microenvironment in which they exist.

Metabolomics data of RA synovial fibroblasts have also highlighted the upregulation of the pentose phosphate pathway which is involved in promoting cell proliferation, invasiveness and inhibition of apoptosis, thereby contributing to pannus formation (Fig. 2) [37].

Similarly to activated myofibroblasts, RA synovial fibroblasts have also shown a dependency on glutamine metabolism, with GC/MS metabolomics showing increased glutamine metabolism and PCR analysis showing increased expression of glutaminase‐1 (GLS1) [38]. Glutamine deprivation resulted in reduced RA synovial fibroblast proliferation and treatment with small molecule inhibitor compound 968 (5‐(3‐bromo‐4‐(dimethylamino)phenyl)‐2,2‐dimethyl‐2,3,5,6‐tetrahydrobenzo[a]phenanthridin‐4(1H)‐one) ameliorated autoimmune arthritis and reduced the number of synoviocytes in SKG mice [38].

TLR2 receptors are capable of inducing mitochondrial mutations in RA synovial fibroblasts, inducing ROS generation, increasing lipid peroxidation and changing the ultrastructure of RA synovial fibroblast mitochondria [39]. Furthermore, TLR2 activation was capable of reducing mitochondrial respiration and attenuating ATP synthesis [39]. However, inhibiting the glycolytic pathway, using small molecule inhibitor 3‐(3‐pyridinyl)‐1‐(4‐pyridinyl)‐2‐propen‐1‐one (3PO), resulted in reducing TLR2‐induced RA synovial fibroblast invasion and migration and attenuated pro‐inflammatory cytokines IL‐6, IL‐8, MCP‐1 and RANTES production [39]. 3PO also reduced the intracellular concentration of lactate, another key metabolite which induces IL‐17 production in CD4+ T cells in RA [40, 41].

### Fibroblasts and fibrosis

Fibroblast‐mediated ECM remodelling is essential for normal tissue structural integrity. However, both trauma and inflammation are known to alter ECM homeostasis. In chronically inflamed tissues, cytokines such as TNFα, IFNy and TGF‐β regulate the expression of ECM proteins [42], which have been shown to alter the inflammatory status of fibroblasts. For example, Tenascin C, an ECM protein that is generated de novo in chronic inflammatory conditions, interacts with TLR4 receptors in macrophages and synovial fibroblasts of RA patients [42].

Critically, a recent study by Zhao *et al*., [43] found that dermal fibroblast metabolism was intricately linked with ECM homeostasis. Genomewide transcriptomic data of human skin fibrosis postradiation therapy compared to age‐matched healthy controls showed a downregulation in oxidative phosphorylation, fatty acid oxidation (FAO) and TCA cycle activity, alongside a concomitant increase in glycolytic processes [43]. Indeed, induction of peroxisome proliferator‐activated receptor signalling using caffeic acid in dermal human primary fibroblasts reduced production of profibrotic proteins and blocking glycolysis using 2‐deoxy‐D‐glucose downregulated ECM protein levels [43].

Research into chronic kidney disease (CKD) has implicated renal fibrosis as a cause of end‐stage renal failure. A key culprit of renal interstitial fibrosis is activated fibroblasts, which aggregate and produce large amounts of ECM components that contribute to progression of CKD [44, 45].

Notably, *in vitro* studies have shown that activated renal interstitial fibroblast stimulated with TGF‐β exhibits decreased mitochondrial respiration and increased expression of glycolytic enzymes HK1,2 and PKM2 as well as increased aerobic glycolysis similar to that observed in dermal fibroblast at sites of skin fibrosis [43, 46]. Similar findings have been demonstrated *in vivo*, using the unilateral ureteral obstruction mouse model of renal interstitial fibrosis, where increased expression of glycolytic enzymes hexokinase (HK) and pyruvate kinase M2 (PKM2) was observed in renal tissue [46, 47].

Fibroblasts are also key culprits in pulmonary fibrosis, a condition which impairs respiratory gas exchange. Lung fibroblasts under healthy conditions remain in a quiescent state, with little collagen production. However, in disease conditions they undergo a major transformation, differentiating into myofibroblasts that exhibit increased proliferation and greater ECM production. Alongside this change in fibroblast phenotype, there is a shift in metabolic status of these activated cells towards aerobic glycolysis, which is dependent on the increased expression of the glycolytic enzyme PFKFB3 [48]. Indeed, it has been shown that inhibiting glycolysis using the PFKFB3 inhibitor 3PO attenuated the differentiation of lung fibroblasts into activated myofibroblasts and reduced their profibrotic phenotype in patients with idiopathic pulmonary fibrosis [48]. The excessive production of ECM components by myofibroblasts has also been achieved *in vitro* by the inhibition of glutaminolysis using glutaminase 1 inhibitors (BPTES), which catabolises the conversion of glutamine into glutamate and then α‐ketoglutarate, suggesting glutamine metabolism regulates increased collagen production in myofibroblast [49].

Excessive collagen deposition in lung fibrosis has also been linked to altered serine and glycine synthesis. TGF‐β, which promotes excessive collagen production in idiopathic pulmonary fibrosis, was found to induce the expression of de novo serine synthesis enzymes phosphoglycerate dehydrogenase (PHGDH), phosphoserine aminotransferase 1 and phosphoserine phosphatase, as well as the expression of the de novo glycine synthesis enzyme serine hydroxymethyltransferase 2 (SHMT2) in primary lung fibroblasts, which are required for collagen synthesis [50]. Indeed, IPF patient lung tissues were found to exhibit increased expression of PHGDH and SHMT2 [50].

Another disease state associated with fibrosis is cardiopathy, in which activated myofibroblasts also play a central pathological role. The development of cardiac fibrosis is similar to the development of fibrosis in fibrotic tissue disorders of the lung, kidney and liver. ECM accumulation impairs cardiac function by increasing the stiffness of tissue and inducing pathological signalling within cardiomyocytes as well as impairing mechano‐electric coupling of cardiomyocytes [51]. Myofibroblasts, as major producers of ECM components, are intimately involved in cardiac fibrosis. Under hypoxic conditions, cardiac fibroblasts increase in proliferation and upregulate alpha smooth muscle (α‐SMA) expression, indicative of a switch to an activated myofibroblast phenotype [52]. Similarly, in an *in vivo* model of ischaemic injury, mice displayed reduced caveolin‐1 and PTEN expression and increased α‐SMA expression in the infarct zone, which was accompanied by increased collagen deposition and reduced cardiac function [52].

Notably, the miRNA, miR‐21, is associated with metabolic alterations in CAFs including increased glycolysis, lactic acid production and expression of LDHA and PKM2 [53]. miR‐21 has been shown to promote cardiac fibroblast to myofibroblast transformation and myocardial fibrosis in a rat model of myocardial infarction [53, 54] and increase cardiac fibrotic remodelling and fibroblast proliferation through the CADM1/STAT3 pathway [55].

Finally, activated myofibroblasts also play a key role in driving fibrosis in liver cirrhosis, a known risk factor for the development of hepatocellular carcinoma (HCC). Accumulation of ECM components at the liver results in fibrotic scarring of liver tissue [56]. Hepatic stellate cells (HSC) are thought to be the primary ECM‐producing cells that, following continuous damage, are transformed into activated myofibroblasts expressing α‐SMA and ultimately are the primary source of CAFs in the development of HCC [47, 57]. In brief, the damage‐induced death of hepatocytes and cholangiocytes epithelial cells of the bile duct results in the activation of HSCs by pro‐inflammatory cytokines released by immune cells [58], which leads to activated myofibroblast that drives hepatic fibrogenesis [59] and CAFs that promote the proliferation and migration of cancer cells in HCC [58].

These hepatic myofibroblasts, like in other fibrotic conditions, secrete large amounts of ECM components including collagen I and III and accumulate fibronectin, elastin and proteoglycans [56], and have been found in both human HCC and murine models of HCC [47, 57]. Therefore, the aforementioned metabolic dysregulation associated with activated myofibroblast in cardiac and pulmonary fibrosis likely plays an important role in the development of liver fibrosis and HCC.

## Fibroblasts and immune cell crosstalk

One aspect that likely facilitates the ability of fibroblasts to regulate inflammatory microenvironments is their interaction with immune cells. In particular, the role that CAFs play in tumour promotion has undergone a conceptual shift with recent research highlighting their contribution to immune suppression and chronic inflammation due to their ability to recruit and interact with resident or circulating immune cells. For example, IL‐6 secretion by a subset of pancreatic CAFs (known as pancreatic stellate cells) promotes the differentiation of immune cells into functional myeloid‐derived suppressor cells (MDSCs) [60]. Furthermore, the release of the chemoattractant M‐CSF, SDF‐1 and MCP‐1 from these pancreatic CAFs promotes the accumulation of MDSCs to the tumour site [60].

CAFs also drive the recruitment of monocytes to the tumour site and their differentiation to the M2 macrophage phenotype which contribute to the tumour progression through secretion of pro‐inflammatory cytokines such as IL‐6, IL‐8, IL‐10 and TGF‐β [61]. Similarly, FAP+ CAFs like mesenchymal stem cells in oesophageal squamous cell carcinoma are thought to promote the migration of macrophages via the secretion of CCL2, IL‐6 and CXCL8 [62].

CAFs have also been shown to impact and modulate adaptive immune cell function and activation. For example, secretion of IL‐6 and TGF‐β from αSMA+ CAFs attenuates dendritic cell (DC) function, maturation and trafficking, thus impacting on T‐cell activation and promoting T‐cell anergy [61, 63]. Furthermore, it has been shown that CAF TGF‐β1 signalling results in tolerogenic DCs that promote the differentiation of T cells towards a regulatory phenotype thereby impairing the antitumour immune response [64]. CAFs in HCC have also been shown to shape the metabolism of regulatory DCs via the upregulation of indoleamine 2,3‐dioxygenase, resulting in reduced T‐cell proliferation and the promotion of regulatory T cells (Tregs) [65]. In breast tumours, FAP+ PDPN+ CAFs are located in close proximity to T cells and are reported to suppress the proliferation of T cells via nitric oxide synthase [66]. In a prostate cancer model, lactate released from glycolytic CAFs was associated with increased Tregs and promoted naive T‐cell polarisation via NF‐kB activation and Foxp3 expression, suggesting CAFs are also capable of shaping the metabolic environment to promote regulatory T‐cell presence at the tumour site [67].

In fibrotic environments, activated myofibroblasts promote the migration of nonresident immune cells by inducing inflammation and by facilitating the restructuring of the ECM proteins to assist immune cell infiltration to these fibrotic sites. In a bleomycin‐induced mouse pulmonary fibrosis fibrotic model, M2 macrophages were found to promote myofibroblast differentiation of lung‐resident mesenchymal stem cells. Furthermore, inhibition of pulmonary M2 macrophage infiltration attenuated the lung fibrosis [68]. Interestingly, biopsies from patients with various forms of kidney disease show that macrophages themselves undergo macrophage to myofibroblast transition in active fibrotic lesions, thereby suggesting macrophages themselves can transdifferentiate to active myofibroblast and contribute directly to renal fibrosis [69]. In addition, T cells have been shown to promote myofibroblast proliferation and contribute to the chronic inflammatory state of inflammatory bowel disease [70], where intestinal fibrosis is in part due to excessive myofibroblast proliferation and excess ECM deposition.

In arthritis, there is substantial evidence that the inflammatory joint pathology is exacerbated by the interactions between synovial fibroblasts and immune cells. In both OA and RA, the diseased synovial joint tissue contains both innate and adaptive immune cells. RA synovial fibroblasts release chemokines such as CCL2 (MCP‐1) when activated with pro‐inflammatory cytokines, which in turn promotes the migration of monocytes to the synovial joint [71, 72]. Activated RA synovial fibroblasts also express CCL5 (RANTES), which attracts T cells and monocytes [72]. Furthermore, both CCL2 and CCL5 can induce the expression of CXCL8 (IL‐8) in RA synovial fibroblasts, thereby promoting neutrophil chemotaxis.

Synovial fibroblasts have also been shown to promote leucocyte survival and activation. Coculture studies have shown that direct cell‐to‐cell interaction impedes the programmed cell death of T and B lymphocytes in culture [73, 74]. Indeed, RA synovial fibroblasts express IL‐15, IL‐16 and IL‐17, which contribute to CD4+ T‐cell proliferation and activation in the synovial membrane [74]. Furthermore, the expression of B lymphocyte stimulator by RA synovial fibroblasts modulates the differentiation and proliferation of B lymphocytes, leading to the production of autoantibodies and rheumatoid factors [75].

In addition, resting T cells also have the capacity to act as regulators of synovial fibroblasts. Resting T cells, with or without T‐cell mitogens, induces the activation of synovial fibroblasts resulting in the upregulation of pro‐inflammatory cytokines IL‐6, IL‐8 and the putative inflammatory pain mediator prostaglandin PGE_2_ [76]. Likewise, various subsets of T lymphocytes (CD4+, CD8+, CD45RO+ or CD45RA+) are capable of activating synovial fibroblasts to varying degrees, resulting in the increased production of inflammatory cytokines by the fibroblasts. This intercellular feedforward loop, derived from cell–cell contact and paracrine signalling between synovial fibroblasts and T lymphocytes, may result in a perpetual cycle of synovial inflammation, which contributes to the chronic inflammatory state observed in RA [77].

## Fibroblasts and senescence

Senescence is a molecular program that creates a state of irreversible growth arrest and can be induced by a wide variety of cellular stress mechanisms. Senescent cells accumulate in ageing tissue and in a wide variety of disease pathologies such as RA [78], tumours [79] and fibrosis [80], and they have been shown to be capable of modulating these disease pathologies in animal models [81]. For example, senescent fibroblasts promote cellular proliferation in the tumour microenvironment, inducing the growth of malignant mammary epithelial cells via the secretion of CXCL1, promoting prostate epithelial cell hyperproliferation via amphiregulin secretion [82] and regulating prostate tumour progression in xenograft models via the production of through secretion of connective tissue growth factor [83]. In RA, senescent fibroblast has been shown to accumulate within the synovial tissue. *In vitro* studies have demonstrated that senescence of RA fibroblasts can be induced by exposure to TNFα, which is prevalent within the joints of RA patients. Furthermore, such senescent RA fibroblasts secrete high levels of pro‐inflammatory cytokines including IL‐6, CCL2 and CXCL8 as well as degradative MMPs [78].

A central feature of senescent cells is their secretory phenotype, termed the senescence‐associated secretory phenotype (SASP). Human fibroblasts, similarly to other cell types that undergo senescence, exhibit a pro‐inflammatory SASP, with increased secretion of pro‐inflammatory cytokines and chemokines [84], which has been implicated in mediating the pathophysiology of inflammaging and chronic inflammatory fibrotic disorders. However, importantly, several studies have provided evidence that this inflammatory SASP is accompanied by an altered metabolic phenotype.

*In vitro*, senescence in fibroblasts can be induced via gamma radiation, proliferative exhaustion (PE) or induction of oncogenes or ROS production, all of which have been shown to be accompanied by changes in fibroblast metabolism. In James *et al*. (2015), senescent fibroblasts induced by either PE or by gamma radiation were found to exhibit overlapping metabolic profiles. Extracellular secretome metabolic profiles showed increased production of citrate, tryptophan metabolism, phospholipid and nucleotide catabolism, whilst intracellular metabolites revealed increased glycolysis, gluconeogenesis, pentose phosphate pathway and pyruvate dehydrogenase kinase (PDK) expression [85]. Furthermore, the change in the fibroblast metabolome is reported to be a dynamic process [86].

Oncogene‐induced senescence in fibroblasts has also been shown to be accompanied by changes in cellular metabolism. For example, Ras oncogene‐induced senescence in the lung fibroblast cell line IMR‐90 was associated with metabolic changes including reduced lipid synthesis, increased FAO and a higher rate of basal oxygen consumption [81], whilst the suppression of PDK1 and increased pyruvate TCA cycle flux has implicated pyruvate dehydrogenase has a central mediator of BRAF oncogene‐induced senescence in human dermal fibroblasts [87]. Notably, pharmacological or genetic inhibition of carnitine palmitoyltransferase 1 (the rate‐limiting step in mitochondrial FAO) was found to reverse metabolic status to presenescence and selectively inhibit the secretory, pro‐inflammatory state associated with oncogene‐induced senescence [81], suggesting that therapeutics that inhibit senescent CAFs could modulate the inflammatory tumour microenvironment.

## Conclusion and therapeutic perspectives

Across different inflammatory disorders and tumours, there is now substantial evidence for the role of fibroblast metabolism in underpinning the pathophysiology of inflammatory microenvironments. Such findings clearly have implications for our understanding of how the inflammatory response is regulated and thus the pathways and processes that drive tumour and chronic inflammatory disease progression.

The switch to an activated state within fibroblasts is accompanied by a shift in metabolic status of the cell and is observed across multiple pathologies including in RA synovial fibroblasts, CAFs and myofibroblasts in fibrotic disorders, suggesting commonality in the pathophysiology of these fibroblast‐associated conditions (Fig. 2). The change from a resting homeostatic state towards the activated state likely necessitates an increased metabolic output in order to drive the altered phenotype of the cell, such as the increased ECM production exhibited in activated myofibroblasts. This change in metabolism is commonly in the form of increased aerobic glycolysis, with activated fibroblasts exhibiting increased expression of glycolytic enzymes in order to drive this increased glycolysis and activated metabolic state, for example increased expression of hexokinase in CAFs and increased expression of PFKFB3 in myofibroblasts, indicative of a high glycolytic activity as a common metabolic signature of activated fibroblasts. Furthermore, increased glutamine metabolism of activated fibroblasts has been reported to be a feature of CAFs, lung myofibroblasts and RA synovial fibroblasts.

Recognition of fibroblasts and their metabolism as key players in mediating inflammatory and immune responses opens up a new therapeutic area for the identification and validation of tractable targets for the development of therapeutic entities. Metabolism and the tumour microenvironment are recognised as central hallmarks of cancer [88], and as such, cancer drug discovery research has for some time looked to target aspects of metabolism. For example, compounds targeting MCT1/2 to disrupt lactate transport are currently being evaluated for antitumor efficacy [89]. Furthermore, inhibition of glycolysis in fibroblasts using the small molecule PFKBP3 inhibitor 3PO has shown efficacy in preclinical studies. In RA synovial fibroblasts, 3PO reduced the secretion of pro‐inflammatory cytokines [40], and TLR2‐mediated invasion and migration [39]. Similarly, the same inhibitor attenuated the differentiation of lung fibroblasts into activated myofibroblasts and reduced their profibrotic phenotype in patients with idiopathic pulmonary fibrosis [48]. An alternative approach is to inhibit glutamine metabolism. Targeting stromal glutamine synthetase in tumours has been shown to inhibit tumour growth and metastasis [90]. Furthermore, the glutaminase 1 inhibitor (compound 968) has been shown to reduce the proliferation of RA synovial fibroblasts and to reduce arthritis severity in an autoimmune arthritis murine model [38]. Furthermore, reduced ECM production in myofibroblasts has been demonstrated using the glutaminase 1 inhibitor BPTES [49], suggesting that inhibition of glutamine metabolism could be a therapeutic approach for lung fibrosis. Notably, these studies together with the commonality seen in the dysregulation of several of the metabolic pathways in fibroblasts across different inflammatory pathologies (Fig. 2, Table 1) suggest there may also be the opportunity to repurpose any developed therapeutics.

**Table 1:** Dysregulated metabolic pathways in stromal fibroblasts.

	Metabolic pathways			
	Aerobic glycolysis	Oxidative phosphorylation	Pentose phosphate pathway	Glutamine metabolism
CAFs	Upregulation of HK2 and PFKL [18]	IDH3α is reduced in CAFs resulting in increased glycolysis and decreased oxidative phosphorylation [22]		Inhibiting glutamine synthetase in stroma and glutaminase in cancer cells reduces tumour weight, nodules and metastasis [90]
Activated Synovial Fibroblasts	High levels of PGK1 present in blood and synovial tissue of RA patients and silencing of PGK1 in RA synovial fibroblasts reduced the secretion of IL‐1β and IFN‐γ [34]	Hypoxia in RA synovial fibroblasts results in reduced oxidative phosphorylation and increased glycolysis [36]	GC/TOF‐MS‐based metabolomic profiling of RA synovial fibroblasts shows upregulation of the pentose phosphate pathway [37]	RA synovial fibroblasts show increased expression of GLS1 [38]
Myofibroblasts	Myofibroblasts show increased expression of the glycolytic enzyme PFKFB3 and inhibiting glycolysis using the PFKFB3 inhibitor 3PO reduced the differentiation of lung fibroblasts into activated myofibroblasts [48]	Dermal fibrotic skin and lung fibroblasts stimulated with TGF‐β results in a switch from mitochondrial respiration to aerobic glycolysis [43, 87]		Lung myofibroblasts show significantly augmented glutaminolysis, which is mediated by elevated Gls1 and inhibition of glutaminolysis using glutaminase 1 inhibitor (BPTES), reduced the expression of ECM components [49]
Senescent fibroblasts	Senescent fibroblasts show increased expression of glycolytic enzymes PDK2, PDK3, PDK4 [85]	Metabolomic profiling of intracellular senescent fibroblasts metabolome shows reduced TCA cycle metabolites such as citrate, indicative of a decline in mitochondrial metabolisms [82]	GC/TOF‐MS‐based metabolomic profiling of senescent fibroblasts conditioned media show reduced TCA activity and shift energy production towards glycolysis, gluconeogenesis, and the pentose phosphate pathway [85]	Metabolomics analysis shows glutamate levels increase in senescent fibroblasts [86]

Despite these promising studies, one of the key challenges in the metabolism therapy area is the identification of targets within metabolic pathways that are both tractable and can be targeted specifically. To this end, the identification of targets that specifically mediate fibroblast metabolism and/or their interaction with immune cells could be a rewarding therapeutic strategy. Such an approach requires further investigation into the metabolic pathways that are altered in activated diseased fibroblasts and the key genes that mediate these pathways. An additional challenge is the heterogeneity of fibroblasts, with several studies having demonstrated that specific fibroblast subsets in RA, fibrosis and cancer can mediate inflammation and/or crosstalk with immune cells. However, the existence of specific subsets also represents the opportunity to therapeutically target specific pathotypes. For example, single‐cell sequencing recently identified anatomically discrete and transcriptomically distinct fibroblast subsets in RA that mediated inflammation and tissue damage [91]. Studies to determine differences in the metabolism of these different fibroblast pathotypes could identify more specific tractable targets for the development of therapeutics.

## Conflict of interest

The authors declare no conflict of interest.

## Author contributions

HF, SPY, CM and SWJ wrote the review and approved it for publication.
